# Psychometric properties of the Czech Integrated Palliative Outcome Scale: reliability and content validity analysis

**DOI:** 10.1186/s12904-020-00552-x

**Published:** 2020-03-25

**Authors:** Karolina Vlckova, Eva Hoschlova, Eva Chroustova, Martin Loucka

**Affiliations:** 1 Center for Palliative Care, Dykova 15, 110 00 Prague, Czech Republic; 2 First Faculty of Medicine, Department of Psychiatry, Charles University, Prague, Czech Republic; 3 Faculty of Arts, Department of Psychology, Charles University, Prague, Czech Republic

**Keywords:** IPOS, Outcome measurement, Validity, Reliability, Patient-reported outcome measure, Palliative care, Symptom assessment, Psychometrics

## Abstract

**Background:**

Outcome measurement is an essential part of the evaluation of palliative care and the measurements need to be reliable, valid and adapted to the culture in which they are used. The Integrated Palliative Outcome Scale (IPOS) is a widely used tool for assessing personal-level outcomes in palliative care. The aim of this study was to provide Czech version of IPOS and assess its psychometric properties.

**Methods:**

Patients receiving palliative care in hospice or hospitals completed the IPOS. The reliability of Czech IPOS was tested with Cronbach alpha (for internal consistency), the intraclass correlation coefficient for total IPOS score and weighted Kappa (for test-retest reliability of individual items). Factor analysis was used for elucidating the construct (Exploratory Factor Analysis). Convergent validity was tested with correlation analysis (Spearman correlation) in a part of the sample, who completed also the Edmonton Symptom Assessment System (ESAS) and the Palliative Performance Scale (PPS).

**Results:**

The sample consisted of 140 patients (mean age 72; 90 women; 81% oncological disease). The Cronbach alpha was 0.789; intraclass correlation was 0.88. The correlations of IPOS with ESAS was R = 0.4 and PPS R = − 0.2. Exploratory factor analysis revealed a 2-factor solution on our data. The first factor covers emotional and information needs and the second factor covers physical symptoms.

**Conclusion:**

Czech IPOS has very good reliability regarding both internal consistency and test-retest reliability. Together with an item analysis results, we can conclude that the Czech adaptation of the tool was successful. The convergent validity needs to be assessed on the larger sample and the proposed 2-factor internal structure of the questionnaire has to be confirmed by using CFA.

## Background

The main goal of palliative care is to improve the quality of life of patients suffering from life-threatening illnesses and their families. Therefore, quality-of-life measurements are important for the evaluation of palliative care interventions and the needs of patients or quantifying the change in health status [1]. A wide variety of measurements currently exists and they differ in the number of measured domains, number of items, mode of administration (questionnaire/interview, patient/proxy) and also in the level of validity and reliability [2]. The Palliative Outcome Scale (POS) is one of the tools for comprehensive measurement of the patients´ main symptoms and concerns [3]. POS is widely used in clinical care, audit, research, and training and it was validated in several languages [4, 5]. The POS measures have been used in different patients populations such as patients with cancer, respiratory, heart, renal or liver failure, and neurological diseases [6–10]. POS-S was developed as an addition to POS to be used as a brief tool specifically focused on physical symptoms [11]. There are also specific variations of POS for dementia or renal failure patients, (POS S-Renal, POS S-Multiple Sclerosis, POS S-Parkinson Disease) [5]. IPOS is the youngest instrument from the POS family which merges questions from POS and POS-S as it was requested from clinicians [11]. IPOS consists of 10 questions which cover main symptoms, patient and family distress, well-being, sharing feelings with family, practical concerns and information needs [11].

IPOS was found to have excellent reliability [12–16] and face and content validity was also confirmed in several studies using cognitive interviews [11, 17, 18] Convergent validity has been confirmed for the original and German IPOS [13], Japanese version of IPOS [14] and French IPOS [16]. In many other countries the process of validation is ongoing and all language version which are currently available, such as Portuguese, Polish, Greek etc., can be found online (www.pos-pal.org).This study aims to provide a valid version of IPOS in Czech and to report the psychometric properties of IPOS from this first pilot Czech study. During the standardization, we followed the manual created by authors of POS [19].

## Methods

This was a mixed-method multicenter study conducted in 6 organizations in the Czech Republic (1 home hospice care, 2 hospices facilities and 3 hospitals). Data were obtained by trained clinical staff - nurses or social workers during the inpatient admission or home visit. The inclusion criteria were: being patient of hospice or home hospice care or palliative care team/unit in the hospital and able to give consent to participate. We excluded patients who had cognitive impairment (judged by the clinical team) and who did not understand the Czech language. Patients completed IPOS and a demographic questionnaire on their own or with help from the staff member. When appropriate, patients were asked to complete IPOS twice for testing of reliability. The second measurement was done when it was possible and feasible from the clinical point of view, predominantly during the next appointment. The instructions were to do it after minimum of 3 days.

IPOS consists of 10 questions with 17 items. Question 1 is about the main concerns and has open-ended options. Q2 addresses specific symptoms and there is also a place for adding any additional symptoms (Q2a-c). Q3-Q6 ask about psychological, spiritual, communication and practical concerns but Q6–8 address positive aspects and the direction of possible answers is opposite. Q10 is not scored and asks patients whether they filled IPOS with any help or by themselves. All questions except Q1 have a numerical scale from 0 to 4 and only one response is allowed for each question. The sum score can range from 0 to 68 and is computed from all items except Q1 and Q2a-c.

The Czech version was created clarifying conceptual definition equivalents in Czech followed by forward and backward translation which was done by independent translators as required by the Manual for the cross-cultural adaptation of the POS [19]. The initial Czech version of IPOS was piloted through cognitive interviews with 5 patients and 5 health care providers from hospice and the face validity of the Czech IPOS was confirmed. The final Czech version of IPOS can be found in Additional file 1.

Part of the sample completed the Edmonton Symptom Assessment System or the Palliative Performance Scale for testing the construct validity of IPOS. Only those data collection sites which use ESAS and PPS as part of routine care were asked to provide both data. The Edmonton Symptom Assessment System (ESAS) is another questionnaire assessing the key patients´ symptoms and concerns and is commonly used in Czech hospices. ESAS consists of 10 items measuring physical symptoms and well-being and patients are asked to rate the symptoms severity from 0 to 10 on a numerical scale [20].

Palliative Performance Scale (PPS) is a tool for measuring performance status of patients in palliative care and it is usually recorded by nurses or by physicians with good inter-rater agreement [21]. It was developed from the Karnofsky Performance Scale [22]. It is oriented on physical functions and activities and can be used for prognostication and planning care [23]. Patients’ performance is scored by percentage in 11 categories from fully ambulatory and healthy (100%) to death (0%). The ratings are based on observation of 5 categories: ambulation, level of activity and evidence of disease, ability to self-care, food/fluid intake and state of consciousness [22].

The Ethical Committee of the General University Hospital in Prague approved the study (Protocol Number 51/18 S-IV) and all participants gave written informed consent.

### Statistical analysis

Internal consistency of the IPOS total score was investigated by using Cronbach ‘s alfa. Item difficulty was calculated using item mean and converted to interval < 0;1 > using formula mean-scale min/(scale max-scale min). Part of the sample (13%) completed the IPOS in two different times for confirmation of temporal stability (T1 and T2) with an average range of 15.6 days between the measures (SD = 9.0). Test-retest reliability of the IPOS total score was evaluated for the part of the sample (*N* = 14, see Table 1) using the intraclass correlation coefficient (ICC). An ICC range of 0.4–0.7 was considered moderate and > 0.75 was considered to represent high test-retest reliability [24]. For each of 17 IPOS items, we also computed four metrics of test-retest reliability: level of agreement, level of agreement within one score, quadratic weighted kappa and Spearman correlation. A range of kappa from 0.41 to 0.60 was considered as moderate, 0.61–0.80 as substantial, and 0.81–1 as almost perfect [25, 26].

**Table 1:** Characteristics of the final sample

	Number of patients (%)	Number of patients who completed IPOS twice (%)	Number of patients who completed IPOS and PPS (%)	Number of patients who completed IPOS and ESAS (%)
Age				
Range	27–95 years	55–88 years	49–92 years	49–89 years
Mean (SD)	72.1 (12.98)	70.0 (10.54)	71.4 (11.01)	70.4 (13.03)
18–55	14 (10)	1 (7.1)	5 (12.5)	2 (14.3)
56–65	23 (16.4)	3 (21.4)	7 (17.5)	4 (28.6)
66–75	44 (31.4)	7 (50)	14 (35)	3 (21.4)
76–85	36 (25.7)	2 (14.3)	9 (22.5)	2 (14.3)
> 85	23 (16.4)	1 (7.1)	5 (12.5)	3 (21.4)
Sex				
Men	50 (35.7)	4 (28.6)	12 (30)	1 (7.1)
Women	90 (64.3)	10 (71.4)	28 (70)	13 (92.9)
Marital status				
Single	16 (11.4)	2 (14.3)	2 (5)	1 (7.1)
Married	52 (37.1)	6 (42.9)	18 (45)	4 (28.6)
Divorced	17 (12.1)	2 (14.3)	5 (12.5)	1 (7.1)
Widowed	54 (38.6)	4 (28.6)	15 (37.5)	8 (57.1)
Registered (homosexual marriage)	1 (.7)	0	0	0
Diagnosis				
Cancer	113 (80.7)	13 (92,9)	39 (97.5)	14 (100)
Other	26 (18.6)	1 (7.1)	1 (2.5)	0
Not available	1 (.7)	0	0	0
Place of care				
Hospice	57 (40.7)	6 (42.9)	20 (50)	14 (100)
Home hospice care	23 (16.4)	5 (35.7)	20 (50)	0
Hospital	60 (42.9)	3 (21.4)	0	0
**Total**	**140**	**14**	**40**	**14**

To test the influence of gender, place of care and age, we used parametric methods (t-test and Pearson correlation coefficient respectively) based on a sufficiently large sample and normal distribution of overall IPOS score.

Moreover, we used factor analysis to explore the possible dimensions of the Czech IPOS questionnaire and to elucidate the constructs. We applied Exploratory factor analysis (EFA) using principal axis factoring as the extraction method and Varimax rotations. The number of factors to be extracted derived from the combination of Kaiser’s criterion and Cattell’s scree plot method.

The Spearman correlations between the IPOS score and two other measures commonly used in palliative care (ESAS and PPS) were assessed to report preliminary results of convergent validity. We expected mid-range correlation between total IPOS score and ESAS total score and PPS (0.5–0.7) because these methods do not cover spiritual, practical and family issues similarly like Murtagh and her colleagues [13]. The non-parametric method was chosen due to quite small sample sizes.

All missing values were excluded from the analysis. A significant *p*-value was set at 0.05. All analyses were conducted within SPSS v. 25.0 (IBM Corp., Armonk, NY, USA).

## Results

### Sample

From November 2017 until August 2018, we collected IPOS data from 144 patients. However, 4 patients had to be excluded from the final sample because they did not complete full IPOS. Most of them were inpatients, only in 16% of patients the place of care was at home provided by the home hospice. The number of patients from the hospital and hospice were similar (43% vs 57%). In the sample, there were few more women (64%) and most of the patients suffered from oncological disease (81%). The detailed description of the sample is in Table 1. Most of the patients (88.6%) needed help in the completion of IPOS.

Table 2 presents descriptive statistics of all 17 IPOS items for the whole sample. We used the short names in the description of items, similarly as Sakurai et al. [14] and Sandham et al. [15] [14, 15]. As a part of the item analysis, we evaluated each item’s difficulty and correlation with the total IPOS score (item-total correlation). The minimum item difficulty was 0.13 (Vomiting), the maximum was 0.6 (Poor mobility). All item-total correlations were higher than 0.3, the highest predictor of the total score was item measuring Weakness with item-total correlation 0.66.

**Table 2:** Description of IPOS items

Item	% response for each value score					M	SD	Mo	Item Difficulty	Item-total correlation
	0	1	2	3	4					
Pain	22.1	25.7	28.6	20	3.6	1.6	1.1	2	0.39	0.48
Shortness of Breath	51.4	19.3	10.7	14.3	4.3	1.0	1.3	0	0.25	0.32
Weakness	10	12.9	32.1	38.6	6.4	2.2	1.1	3	0.55	0.66
Nausea	53.6	22.9	12.1	9.3	2.1	0.8	1.1	0	0.21	0.46
Vomiting	74.3	9.3	9.3	6.4	0.7	0.5	1.0	0	0.13	0.37
Poor Appetite	28.6	17.1	24.3	26.4	3.6	1.6	1.3	0	0.40	0.58
Constipation	46.4	17.9	13.6	20.7	1.4	1.1	1.2	0	0.28	0.44
Sore Mouth	26.4	23.6	21.4	26.4	2.1	1.5	1.2	0	0.39	0.33
Drowsiness	18.6	17.1	37.1	25	2.1	1.8	1.1	2	0.44	0.48
Poor Mobility	10.7	9.3	22.9	43.6	13.6	2.4	1.2	3	0.60	0.49
Anxiety	32.1	14.3	32.1	15.7	5.7	1.5	1.2	0	0.37	0.58
Family Anxiety	10.7	10	30.7	32.1	16.4	2.3	1.2	3	0.58	0.50
Depression	40.7	16.4	31.4	9.3	2.1	1.2	1.1	0	0.29	0.50
Feeling at Peace	15	36.4	28.6	15	5	1.6	1.1	1	0.40	0.59
Share Feelings	30.7	29.3	16.4	17.9	5.7	1.4	1.3	0	0.35	0.44
Information	51.4	28.6	12.1	5.7	2.1	0.8	1.0	0	0.20	0.48
Practical Problems	52.9	20	17.1	7.1	2.9	0.9	1.1	0	0.22	0.45

*M*  mean, *SD* standard deviation, *Mo* modus.

### Influence of gender, age and place of care

The total IPOS score did not differ for men and women (*t* = − 1.537, *p* = 0.127) nor did it correlate with the age of patients (*r* = 0.141, *p* = 0.096). However, we found a significant difference in the total IPOS score when comparing patients from hospices and patients from hospitals (*t* = − 3.613, *p* < 0.001). More specifically, the average total IPOS score of patients from hospices was lower (38.75, *SD* = 9.11) than the average score of patients from hospitals (44.28, *SD* = 8.77).

### Reliability

Cronbach’s alpha for 17 IPOS items (which are used for calculation of the overall score) was 0.789. Temporal stability was evaluated for all items separately as well as for the overall score. A one-way intra-class correlation coefficient of IPOS total score indicated a high level of temporal stability (*ICC* = 0.88, *95% CI:* 0.56–0.94). Sufficient test-retest reliability was also supported by significant Spearman correlation between two total IPOS scores in T1 and T2 (*r* = 0.88, *p* < 0.05). For most of the items significant Spearman correlations were found as well as fair to good levels of weighted kappa, however, several items showed rather low temporal stability, mainly items called Family anxiety, Practical problems, Drowsiness or Anxiety. For more detailed results, please see Table 3.

**Table 3:** Temporal stability

	T1	T2	Agreement			
	Mean (SD)	Mean (SD)	Agreement (%)	Agreement within one score (%)	Weighted kappa (95% CI)	Spearman correlation
Pain	1.6 (1.3)	1.4 (1.0)	35.7	92.9	0.66 (0.40–0.92)	0.69^b^
Shortness of Breath	1.0 (1.2)	1.4 (1.5)	57.1	78.6	0.60 (0.21–0.99)	0.62^a^
Weakness	1.5 (1.0)	1.9 (1.2)	50.0	78.6	0.54 (0.18–0.91)	0.54^a^
Nausea	0.9 (1.1)	0.6 (0.9)	35.7	92.9	0.59 (0.41–0.77)	0.49
Vomiting	0.7 (1.1)	0.4 (0.8)	64.3	85.7	0.58 (0.29–0.86)	0.77^b^
Poor Appetite	1.1 (1.3)	1.6 (1.3)	42.9	92.9	0.65 (0.31–0.99)	0.67^b^
Constipation	0.9 (1.2)	0.9 (1.2)	71.4	71.4	0.46 (−0.02–0.93)	0.51
Sore Mouth	1.6 (1.3)	1.5 (1.0)	57.1	92.9	0.60 (0.15–1.05)	0.63^a^
Drowsiness	1.1 (1.1)	1.9 (0.9)	7.1	71.4	0.33 (0.06–0.60)	0.43
Poor Mobility	2.1 (1.2)	2.4 (0.9)	42.9	85.7	0.41 (0.03–0.79)	0.53
Anxiety	1.0 (1.3)	1.2 (1.1)	28.6	71.4	0.31 (−0.11–0.72)	0.35
Family Anxiety	2.1 (1.1)	2.6 (0.8)	42.9	71.4	0.02 (−0.33–0.37)	0.53
Depression	0.7 (1.1)	0.5 (0.9)	71.4	92.9	0.74 (0.48–1.01)	0.83^b^
Feeling at Peace	1.1 (0.9)	1.2 (1.1)	57.1	85.7	0.54 (0.12–0.96)	0.50
Share Feelings	1.1 (1.4)	1.2 (1.1)	50.0	92.9	0.77 (0.56–0.98)	0.80^b^
Information	0.2 (0.6)	0.2 (0.4)	71.4	100.0	0.40 (−0.08–0.89)	0.32
Practical Problems	0.1 (0.4)	0.4 (0.7)	71.4	92.9	0.27 (−0.23–0.77)	0.32
IPOS	18.9 (9.8)	21.1 (7.2)	–	–	0.83^c^ (0.56–0.94)	0.88^b^

^a^. Correlation is significant at the 0.05 level (2-tailed)

^b^. Correlation is significant at the 0.01 level (2-tailed)

^c^. One-way Intraclass Correlation Coefficient (ICC)

**Table 4:** Factor loadings

	Factor 1	Factor 2
Anxiety	0.711	0.085
Feeling at peace	0.694	0.128
Depression	0.667	0.019
Information	0.531	0.066
Practical Problems	0.515	0.051
Share Feelings	0.431	0.109
Family Anxiety	0.374	0.258
Shortness of Breath	0.156	0.147
Nausea	0.017	0.607
Vomiting	−0.074	0.588
Poor Appetite	0.204	0.584
Weakness	0.403	0.513
Sore Mouth	−0.084	0.462
Drowsiness	0.173	0.429
Poor Mobility	0.220	0.381
Constipation	0.124	0.376
Pain	0.247	0.344

### Exploratory factor analysis

Both Kaiser-Meyer-Olkin Measure of Sampling Adequacy (0.696) and Bartlett’s test of sphericity (*p* < 0.001) indicated that a factor analysis might be useful with our data. Based on the combination of Kaiser’s criterion and Cattell’s scree plot method, we decided to present the two-factor model (Table 4) as an output of EFA which explains 29.1% of the variance (*Factor 1:* 15.9%, *Factor 2*: 13.3%) and the factors showed a correlation of 0.316.

#### Convergent validity

Spearman’s correlation of the sum score of IPOS and PPS was found to be weaker than was expected by our hypotheses and non-significant (*Rs*(40) = −0.249; *p* = 0.121), correlation with ESAS showed to be on a moderate level (*Rs*(14) = 0.414; *p* = 0.141), however, not significant due to a very small research sample. Data from PPS and ESAS were not available from many patients so these results have to be considered preliminary only.

## Discussion

This study aimed to provide a valid version of the Czech IPOS and to report the psychometric properties of IPOS. Item analysis results showed that the Czech adaptation of the tool was successful. This study showed also that the Czech IPOS has very good reliability regarding internal consistency and we preliminary assessed the validity of the Czech IPOS and temporal stability.

Items analysis showed that all of the items in IPOS meet the requirements for item difficulty and item-total correlation. The lowest discriminant ability was found in item Vomiting because 75% of patients did not report this symptom. This is not consistent with previous results [15]. However, in Sandham et al. study only hospice patients were assessed which might have caused the difference [15]. Another study with patients from hospitals and home-based palliative services found similar results when Vomiting, Practical matters and Having enough information did not have full range of responses [13].

Regarding influence of place, age or gender, in our sample, we found significant differences in the total IPOS score according to the place of care which was also confirmed in other countries for POS [27, 28]. This might be explained by the fact that patients in hospices are usually in the terminal stage of disease with well-controlled symptoms as the median of the length of stay in Czech home hospices is around 10 days [29]. IPOS total score did not differ according to age or gender which is consistent with other studies [15].

The reliability of IPOS was measured in two ways with Cronbach alpha and test-rest reliability. The Cronbach alpha showed a high internal consistency of the Czech version of IPOS which is consistent with other studies [12, 13, 15]. IPOS was completed twice by 14 patients and test-retest reliability was confirmed by a sufficient intraclass-correlation coefficient. Some items showed low temporal stability, mainly items called Family anxiety, Practical problems, Drowsiness or Anxiety (0.02–0.33) which is not consistent with Japanese validation where items with the lowest temporal stability (0.522–0.622) were Share Feelings, Information and Practical Problems, for others items ICC was higher than 0.7 [14]. This study is missing independent global change rating which would confirm stability of patients´ health condition. Condition of patients in palliative care is fast-changing which makes the interpretation of our results more difficult. The low temporal stability of these items in Czech IPOS might be also explained by the fact that time between measurement was longer than in previous studies and varied (*M* = 15.6, *SD* = 9). In other studies retest was conducted the next day [14, 30]. Therefore, we need to confirm the retest reliability for Czech IPOS in a shorter period. On the other hand, the second measurement should be done later than the next day to avoid bias that respondents may recall their previous responses [14]. These results show that Practical Problems is an item on which we should focus our attention because it is unstable, and it can change even within 1 day.

The results of factor analysis showed the two-factor model could be applied to our data. The first factor consists of items associated with psychological concerns (Anxiety, Depression, Information etc.) and the second factor is composed of items assessing physical symptoms. Only the item Shortness of breath cannot be easily assigned to one of these factor groups because the loadings reached the low and almost equal level. Sandham and her colleagues identified unidimensionality in IPOS measuring palliative care needs of patients [15]. Even though our data showed the possibility of applying the two-factor model for Czech IPOS, there is a significant correlation between both factors (*R* = 0.316). In our study, we were limited by the size of the overall sample not sufficient to apply Confirmatory factor analysis (CFA). Murtagh and her colleagues identified three factors in IPOS using CFA – Physical Symptoms, Emotional Symptoms and Communication/Practical Issues [13]. This suggests that subscales could differ according to socio-cultural context or that we need more data for testing our two-factor model and the three-factor model using CFA and to compare which of these models is more precise for our population.

In terms of convergent validity, the overall score was correlated with PPS which is a tool measuring physical status [22] and the correlation was weaker than expected because this tool is only focused on physical symptoms. For correlation with ESAS, we found a moderate correlation which was not significant because of the small number of patients who completed IPOS and ESAS. Correlation with ESAS was also confirmed in other study [13]. Sakurai and his colleagues also confirmed validity of IPOS using other instruments (EORTC QLQ-30, FACIT-Sp12, and STAS) and found strong to moderate correlations, except for the item Information [14]. One possible explanation is that this item is rather unique as the only similar question from STAS is answered by a clinician [14]. Correlation of APCA African POS and MVQoLI were found to be weak to moderate for which the explanation might be that different measures of quality of life use different conceptualizations of this term [30].

## Limitations

This study has several limitations. We found moderate but not significant correlation of IPOS and ESAS which means that we cannot confirm convergent of validity of Czech IPOS due to small sample who completed IPOS and ESAS. These results only imply trend which was confirmed in other studies. Due to logistical demand on participating staff it was not possible to get ESAS from every patient in the sample. Only those data collection sites which use ESAS and PPS provided both data. We also could not conduct confirmatory factor analysis on this data due to insufficient sample size. The interval of retest should be shorter with a low level of variability or instead of short time period we should use external criterion to judge stability of patients´ condition. The number of patients who completed the second measurement in this study was very low, therefore, more data for more precise retest reliability results are needed.

## Conclusion

This study confirmed that the Czech version of IPOS might be used in the clinical setting and the cultural adaptation was successful. This study also further proved that IPOS is a reliable method for assessing the quality of life of patients in palliative care.

## Supplementary information

**Additional file 1.** Czech version of IPOS

## Data Availability

The datasets used during the current study are available from the corresponding author on reasonable request.

## Abbreviations

POS: Palliative outcome scale
IPOS: Integrated palliative outcome scale
ESAS: Edmonton symptom assessment system
PPS: Palliative performance scale
ICC: Intraclass correlation coefficient
EFA: Exploratory factor analysis
CFA: Confirmatory factor analysis
APCA: African palliative outcome scale
MVQoLI: Missoula-Vitas quality of life index
EORTC QLQ-30: European organisation for research and treatment of cancer quality of life questionnaire
FACIT-Sp12: Functional assessment of chronic illness therapy – spiritual well-being
STAS: Support team assessment schedule
