# Supplementary material for: Psychometric properties of the Czech Integrated Palliative Outcome Scale: reliability and content validity analysis
Source: BMC Palliat Care. 2020 Mar 25;19:39. doi: 10.1186/s12904-020-00552-x (PMC7098098; doi:10.1186/s12904-020-00552-x)
Supplement: Supplementary file 1 — Additional file 1. Czech version of IPOS [file 12904_2020_552_MOESM1_ESM.pdf]

Appendix 1

Pro personál – Číslo pacienta:

IPOS
www.pos-pal.org
Formulář pro personál

In collaboration with
CENTRUM PALIATIVNÍ PÉČE
CENTER FOR PALLIATIVE CARE

Jméno a Příjmení: ....

Datum: (dd/mm/rrrr) __ __ / __ __ / __ __ __ __

Otázka 1. – Jaké byly pacientovy hlavní problémy či obtíže **<u>v posledních 3 dnech</u>**?

**1a.**	
**1b.**	
**1c.**	

Otázka 2. – Pro každou obtíž, prosím, zaškrtněte jedno políčko, které nejlépe vystihuje, jak byl pacient ovlivněn touto obtíží během posledních 3 dnů.

	Vůbec	Mírně	Středně	Silně	Nesnesitelně
**Bolest**	0 ☐	1 ☐	2 ☐	3 ☐	4 ☐
**Dušnost**	0 ☐	1 ☐	2 ☐	3 ☐	4 ☐
**Slabost či nedostatek energie**	0 ☐	1 ☐	2 ☐	3 ☐	4 ☐
**Nevolnost (pocit na zvracení)**	0 ☐	1 ☐	2 ☐	3 ☐	4 ☐
**Zvracení**	0 ☐	1 ☐	2 ☐	3 ☐	4 ☐
**Nechutenství**	0 ☐	1 ☐	2 ☐	3 ☐	4 ☐
**Zácpa**	0 ☐	1 ☐	2 ☐	3 ☐	4 ☐
**Bolesti či sucho v ústech**	0 ☐	1 ☐	2 ☐	3 ☐	4 ☐
**Ospalost**	0 ☐	1 ☐	2 ☐	3 ☐	4 ☐
**Snížená pohyblivost**	0 ☐	1 ☐	2 ☐	3 ☐	4 ☐

Zapište, prosím, jakékoliv **<u>jiné obtíže neuvedené výše a zaškrtnutím jednoho políčka</u>** označte, jak pacienta tyto obtíže v posledních 3 dnech ovlivnily.

	Vůbec	Mírně	Středně	Silně	Nesnesitelně
**2a**	0 ☐	1 ☐	2 ☐	3 ☐	4 ☐
**2b**	0 ☐	1 ☐	2 ☐	3 ☐	4 ☐
**2c**	0 ☐	1 ☐	2 ☐	3 ☐	4 ☐

V posledních třech dnech:

	Vůbec	Výjimečně	Občas	Většinu času	Pořád
**Otázka 3.** Cítili jste úzkost nebo obavy z vaší nemoci nebo léčby?	0 ☐	1 ☐	2 ☐	3 ☐	4 ☐
**Otázka 4.** Cítil někdo z vaší rodiny úzkost nebo obavy o vás?	0 ☐	1 ☐	2 ☐	3 ☐	4 ☐
**Otázka 5.** Cítil jste se depresivně?	0 ☐	1 ☐	2 ☐	3 ☐	4 ☐

	Pořád	Většinu času	Občas	Zřídka	Vůbec
**Otázka 6.** Pociťujete vnitřní klid?	0 ☐	1 ☐	2 ☐	3 ☐	4 ☐
**Otázka 7.** Byli jste schopni hovořit se svou rodinou a přáteli dostatečně o tom, jak se cítíte?	0 ☐	1 ☐	2 ☐	3 ☐	4 ☐
**Otázka 8.** Dostáváte tolik informací, kolik si přejete mít?	0 ☐	1 ☐	2 ☐	3 ☐	4 ☐

	Problémy řešeny / žádné problémy	Problémy většinou řešeny	Problémy částečně řešeny	Problémy spíše neřešeny	Problémy vůbec neřešeny
**Otázka 9.** Byly řešeny praktické problémy (finanční, osobní atd.) vyplývající z vašeho onemocnění?	0 ☐	1 ☐	2 ☐	3 ☐	4 ☐

	Sám/a	S pomocí někoho z přátel či příbuzných	S pomocí někoho z ošetřujícího personálu
**Otázka 10.** Jak jste vyplnil/a tento dotazník?	☐	☐	☐

Pokud ve vás některé z otázek položených v dotazníku vyvolávají obavy, promluvte si, prosím, s někým z lékařů či sester.
